# Apnea causes microvascular perfusion maldistribution in isolated rat lungs

**DOI:** 10.14814/phy2.14085

**Published:** 2019-05-03

**Authors:** Robert L. Conhaim, Kal E. Watson, Oleg Broytman, Mihaela Teodorescu

**Affiliations:** ^1^ The William S. Middleton Memorial Veterans Hospital Madison Wisconsin; ^2^ Department of Surgery School of Medicine and Public Health University of Wisconsin Madison Wisconsin; ^3^ Department of Medicine School of Medicine and Public Health University of Wisconsin Madison Wisconsin

**Keywords:** Blood flow, microspheres, pulmonary microcirculation, pulmonary vascular biology, sleep apnea

## Abstract

Obstructive sleep apnea is associated with significant cardiovascular disease, yet little is known about the effects of OSA on pulmonary microvascular perfusion. In a recent report, we showed that pulmonary microvascular perfusion was significantly mal‐distributed in anesthetized, spontaneously breathing rats exposed to five episodes of obstructive apnea. We quantified microvascular perfusion by analyzing trapping patterns of 4 *μ*m diameter fluorescent latex particles infused into the pulmonary circulation after the last episode. We could not determine if the perfusion maldistribution was due to the effects of large subatmospheric intrapleural pressures during apnea, or to precapillary OSA hypoxic vasoconstriction. To address this, we repeated these studies using isolated, buffer‐perfused rat lungs (*P*_pulm art_, 10 cm H_2_O) ventilated in a chamber (−5 to −15 cm H_2_O, 25 breaths/min; *P*_trachea_ = 0). We simulated apnea by clamping the trachea and cycling the chamber pressures between −25 and −35 cm H_2_O for five breaths. After five apnea episodes, we infused 4 *μ*m diam. fluorescent latex particles into the pulmonary artery. The number of particles recovered from the venous effluent was 74% greater in nonapneic isolated lungs compared to apneic lungs (*P* ≤ 0.05). Apneic lungs also had perfusion maldistributions that were 73% greater than those without apnea (*P* ≤ 0.05). We conclude that simulated apnea in isolated, perfused rat lungs produces significantly greater particle trapping and microvascular perfusion maldistribution than in nonapneic isolated lungs. We believe these effects are due to the large, negative intrapleural pressures produced during apnea, and are not due to hypoxia.

## Introduction

Obstructive sleep apnea (OSA) is a significant public health problem affecting an estimated 20 million people in the United States (Young et al. 1993). OSA is associated with significant cardiovascular disease, including pulmonary and systemic hypertension, arrhythmias, heart failure, and possibly pulmonary embolus (Somers et al. 2008; Berghaus et al. 2016; Floras 2018). Episodes of OSA transiently reduce cardiac output and raise pulmonary arterial pressure, implying that pulmonary blood flow is reduced during these episodes (Floras 2018). OSA can transiently reduce intrathoracic pressure to as low as – 60 mmHg, and also produce hypoxia both of which may interrupt pulmonary blood flow (Dempsey et al. 2010).

Little is known about how these factors affect pulmonary microvascular perfusion during apnea. In a recent report, we showed that pulmonary microvascular perfusion was significantly maldistributed in anesthetized, spontaneously breathing rats exposed to obstructive apnea (Watson et al. 2012). We produced apnea by clamping each rat's tracheal cannula for 10 breaths after which it was released to allow the animals to again breathe spontaneously. We repeated this each 20 sec for a total of 5 apneic episodes. We quantified microvascular perfusion by infusing 4 *μ*m diam. fluorescent latex particles into the pulmonary circulation after the last apnea episode. We then statistically analyzed the particle trapping patterns in confocal images of the lungs using methods we developed previously (Conhaim et al. 2003). As in human OSA, it was not possible for us to determine if the perfusion maldistribution we found was due to the deeply negative intrapleural pressures or to hypoxic pulmonary vasoconstriction during apnea.

To address this, we repeated these studies using isolated, perfused rat lungs. If we found similar perfusion maldistribution in isolated lungs, it would suggest that negative intrapleural pressures had a role in causing perfusion maldistribution.

## Methods

The methods we used were approved by the Animal Care and Use Committee of our institution. Before use, the animals were housed, two animals per cage, in nonsterile rodent microisolater cages with filtered cage tops.

We isolated lungs from retired, male, breeder Sprague‐Dawley rats (450–550 g), which we chose because of their relatively large size. We anesthetized the rats using isoflurane and sacrificed them by exsanguination following heparin infusion (750 U/kg).

### Lung preparation

We opened the chest, cannulated the trachea, pulmonary artery and left atrium using polyethylene tubing (PE 200), removed the heart and lungs *en bloc*, and placed them dorsal side down into a sealed Plexiglas chamber where they were warmed by an incandescent lamp that produced lung surface temperatures of 33 ± 2°C.

**Figure 1:**
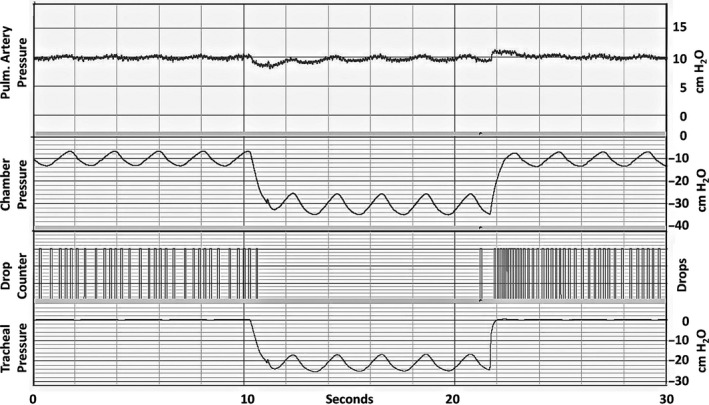
Recordings before, during, and after a typical 5‐breath apnea event. Top‐to‐bottom: pulmonary artery pressure (cm H_2_O), chamber pressure (cm H_2_O), drops of venous outflow, and tracheal pressure (cm H_2_O).

The tracheal, pulmonary artery, and left atrial cannulas were fitted to connectors in the walls of the chamber. The tracheal cannula was open to the atmosphere. The chamber was connected to a vacuum source that set pressure within it to −5 cmH_2_O. The lungs were ventilated by using a piston pump connected to the chamber that cycled the pressures within it between −5 and −15 cmH_2_O at 25 cycles/min. To initiate apneic breathing, an electromagnetic switch (Mouser Electronics, Mansfield, TX) obstructed the tracheal cannula, while a second switch simultaneously changed the source of the chamber vacuum to one set at −25 cmH_2_O. We chose this value because it was similar to the value we measured in esophageal cannulas of anesthetized rats after their tracheal cannulas were clamped to simulate apnea. The piston pump settings remained unchanged. After five breaths, the switches returned to their previous settings which caused the lungs to be ventilated as they were before apneic breathing. We will return to this point below. Recordings of these pressures in a typical apnea are shown in Figure 1. This process was repeated until the lungs was subjected to 5 apneas (5 breaths each), spaced 2 min. apart. After the fifth apnea, latex particles were infused into the pulmonary circulation as described below (*Latex Particle Infusion*).

We perfused the lungs via the pulmonary artery using a buffered hetastarch solution (1.5% *Hespan* in PBS; B. Braun Medical, Inc). The solution was pumped into a reservoir that supplied the pulmonary arterial catheter. The reservoir was equipped with an overflow in which the meniscus at the overflow outlet was set 10 cm above the bottom (dorsal side) of the lung. The perfusate was pumped from a beaker into the reservoir, and the perfusate that did not enter the lung returned to the beaker via the overflow. This set‐up therefore provided a constant pulmonary artery pressure of 10 cm H_2_O. The venous cannula was set level with the dorsal (bottom) side of the lung (*P*_ven_ = 0 cm H_2_O).

We continuously recorded the chamber weights while the lungs were perfused to monitor them for any signs of edema. Any lungs that gained more than 2–3 g, which usually happened suddenly, were considered to be edematous and were discarded. Only data from nonedematous lungs are reported here. Lungs in our present studies were limited to 5 breaths per apnea episode, although we used 10 breaths per episode in our intact rat studies. We found that isolated lungs were more likely to become edematous after 10 apneic breaths, so we limited each episode to 5 breaths in our present studies.

### Latex particle infusion

We prepared the infusion solution by adding 25 *μ*g of bovine serum albumin to 240 *μ*L (1 × 10^8^ particles) of a stock aqueous solution of rhodamine labeled latex particles (*Bangs Laboratories*, Fishers, IN). The albumin coated the particles to prevent them from clumping in the presence of plasma ions. We diluted this solution with 0.25 mL of phosphate buffered saline and infused it into the venous catheter over 30 sec. We infused the particles after the fifth (last) apneic episode to duplicate the infusion conditions we used in our previous study in intact rats (Conhaim et al. 2019b). Our goal was to determine the effects of the preceding apneas on the perfusion distribution. Any such maldistribution, if present, could have implications for pulmonary microvascular perfusion distribution in chronic obstructive apnea during wakefulness.

### Venous sample collection

Drops falling from the venous cannula interrupted the light beam of a drop counter that recorded the perfusate flow rate. The drops were collected in tubes using an automated sample collector that placed a new tube beneath the drop counter every 30 sec. Tube samples were collected from 0.5 to 5.0 min after the start of particle infusion.

When the particle collections were complete, we clamped the arterial and venous cannulas, inflated the lungs to 20 cm H_2_O with compressed air, and maintained the lungs at this pressure for 2 days to allow them to dessicate.

### Venous sample particle counts

We used a flow cytometer (*MACSQuant Analyzer 10*, Miltenyl Biotec, Cologne, Germany) to count the numbers of particles within each of the venous effluent samples collected from each lung. We also sampled the infusion solutions to verify the numbers of particles infused. Latex particle concentrations in venous samples are expressed as a percentage of the total number of particles infused.

### Latex particle perfusion distribution analysis

We employed methods that we developed and have described in detail previously (Conhaim et al. 2003). In brief, we cut a 2–3 mm‐thick sagittal section through the hilum of the dessicated left lung and placed the section onto the stage of a confocal fluorescence microscope (Nikon A1R, Nikon Metrology, Brighton, MI). We obtained 8–10 sequential digital images of the section while it was illuminated with a wavelength of light that caused the latex particles trapped within it to fluoresce. The images were then assembled into a montage of the entire hilar section. Example montages showing latex particles trapped within Control and Apnea lungs appear in Figure 4. From each of these montages, we collected eight randomly distributed images (512 × 512 pixels) of the total hilar section area. Example images are also shown in Figure 4. We then used public domain software (ImageJ 1.51R; http:/imagej.nih.gov/ij) to obtain the *X*–*Y* address of every particle within each of the eight 512 × 512 images obtained from each hilar montage.

We used multiscale statistical analysis to quantify the particle distribution within each of the eight images as described previously (Conhaim et al. 2003). Briefly, we used a Statistical Analysis Software program (SAS Institute, Cary, NC) to divide each 512 × 512 image into quadrants (2 × 2 array), and then used the *x*–*y* address of each particle to determine how many particles were present within each quadrant. The mean (*μ*) and variance (***σ*** ^2^) of the particle counts among the four quandrants were calculated. These were expressed as a ratio (***σ*** ^2^/*μ*), which is known as the *dispersion index* (DI) (Diggle 1983). LogDI was plotted versus the quadrant tissue volume (1680 × 1680 × 100 *μ*m). The image was further subdivided (4 × 4, 8 × 8,…512 × 512; 9 steps), and logDI was plotted against the corresponding tissue volume at each step to produce a dispersion index plot.

In a dispersion index plot, the particle distribution is considered to be statistically random if ***σ*** ^2^ = *μ*. This yields a DI value of one, and a logDI value of zero. The more logDI > 0, the more the particle distribution is considered to be statistically clustered. The more logDI < 0, the more the particle distribution is lattice‐like, as in the distribution of squares on a checker board (Conhaim et al. 2003; Grieg‐Smith 1952).

### Statistical methods

Results are expressed as mean ± SD. A total of six air‐dried lungs were prepared for each treatment group. Statistical comparisons of logDI values were conducted using one‐way analysis of variance and Fisher's Least Significant Difference post hoc test to determine if differences were significant among the 9 logDI steps (2 × 2…512 × 512) in each treatment groups. Differences were considered to be significant at *P* ≤ 0.05.

## Results

### Perfusate flows

**Figure 2:**
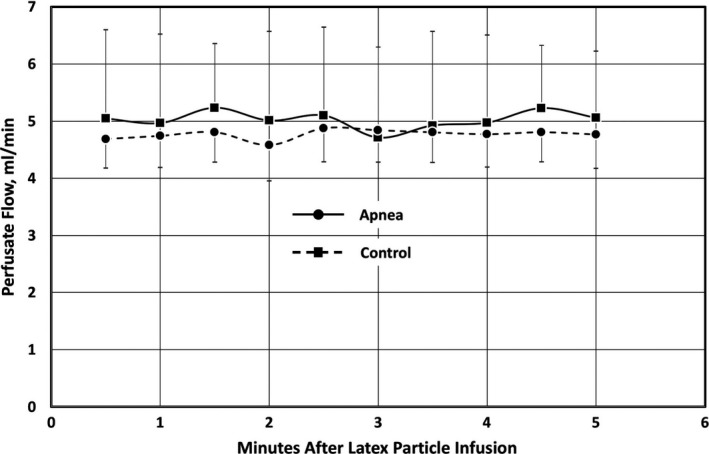
Perfusate flows (mean ± SD) in isolated lungs after latex particles were infused over 30 sec beginning at Time 0. Particle infusion into Apnea lungs (*n* = 6) occurred after completion of the 5th apnea episode. Particle infusions into Control lungs (*n* = 6) occurred after the same duration as in Apnea lungs, although Control lungs were not subjected to apnea.

Initial perfusate flows, measured 15 min after lung perfusion began but before apnea was initiated, averaged 4.6 ± 0.6 mL/min in the Apnea group, and 4.6 ±1.7 mL/min in the Control group (n.s.). Apnea caused flows to stop (Fig. 1, Drops). Flows in both groups after particle infusion, which followed the fifth apnea in the Apnea group, are shown in Figure 2. Average flows for the Apnea group after particle infusion were 4.6 ± 1.1 mL/min, and 5.0 ± 1.3 for the Control group (n.s.). We have shown previously that particle infusion into isolated lungs does not affect flow rate (Watson et al. 2018).

### Venous particle concentrations

**Figure 3:**
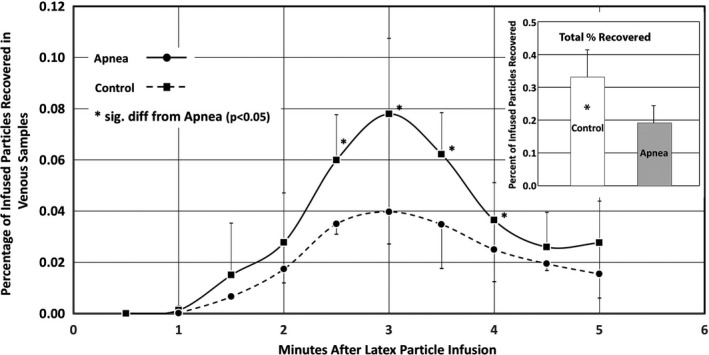
Percentage of infused 4 *μ*m particles recovered in venous effluent samples, and total particles recovered (inset) (mean ±SD). Data were collected from six lungs in each treatment group (Apnea, Control).

Figure 3 shows the concentrations of infused 4 *μ*m particles measured in venous outflow samples collected every 0.5 min from 0.5 to 5 min after the start of particle infusion. Concentrations of particles in samples were significantly higher in Control lungs than in Apnea lungs in samples collected at 2.5, 3.0, 3.5 and 4.0 min after the start of particle infusion. The total number of particles collected from all venous samples averaged 0.33 ± 0.08% of the particles infused into Control lungs compared to 0.19 ± 0.05% of the particles infused into Apnea lungs (*P* ≤ 0.05) (Fig. 3, Inset).

### Latex particle perfusion distribution analysis

**Figure 4:**
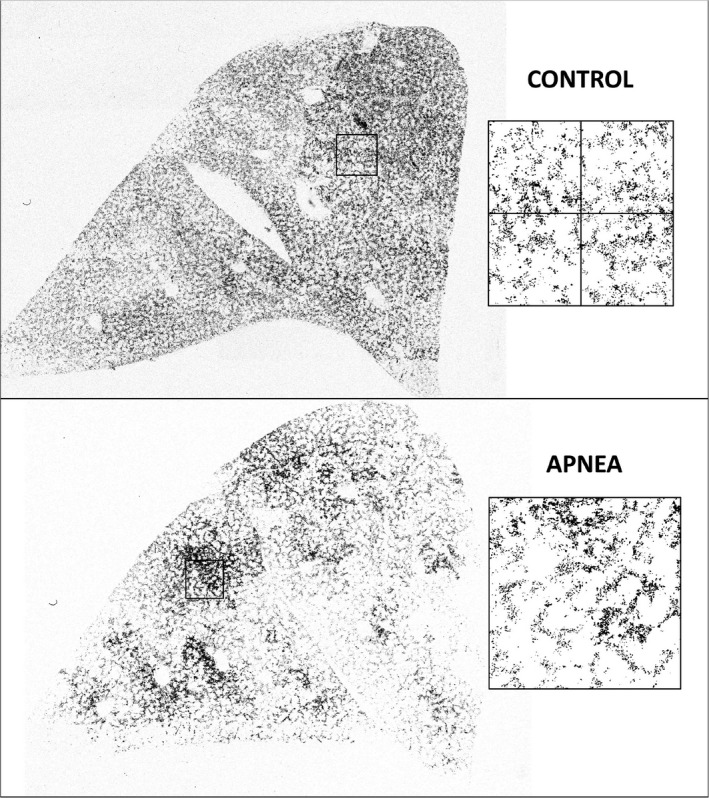
Confocal montages of latex particle trapping patterns in Control and Apneic isolated, perfused lungs. The boxed areas show examples of areas in which latex particle distributions were quantified. The boxes were first subdivided 2 × 2, as illustrated in the Control image. The mean (*μ*) and variance (*σ* ^2^) of the particle counts within each quadrant were calculated and expressed as (*σ* ^2^/*μ*), which is known as the Dispersion Index. The boxes were further subdivided into 4 × 4, 8 × 8, …256 × 256, and the Dispersion Indexes were calculated at each step. A total of 8 such boxes were collected from each lung image, and the nine Dispersion Index steps from each box were averaged to represent the values for that lung. A total of six lungs were imaged and analyzed in each treatment group (Apnea, Control).

**Figure 5:**
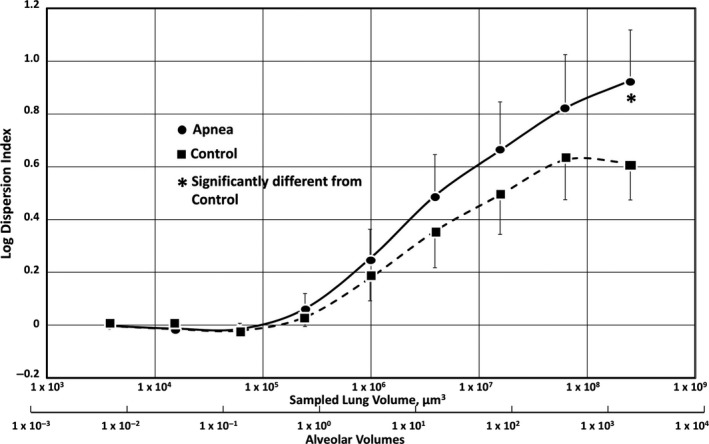
Dispersion index plot of latex particle trapping patterns in the isolated rat lungs subjected to obstructive apnea (circles), and in control lungs not subjected to apnea (squares) (mean ± SD). Apnea alone produced significantly more perfusion maldistribution than controls (*P* ≤ 0.05) at tissue volumes of 1 × 10^3^–1 × 10^4^ alveoli (asterisk). LogDI values at the 8 smaller tissue volumes were not significantly different between Apnea (*n* = 6) and Control (*n* = 6).

It is obvious in Figure 5 that latex particle trapping in Apnea lungs was more maldistributed than in Control lungs. Quantification of these distributions using logDI analysis (Fig. 4) revealed that logDI for Apnea lungs at the largest tissue volume (10^3^–10^4^ alveolar volumes) averaged 0.92 ± 0.19, compared to 0.61 ± 0.14 in Control lungs (*P* ≤ 0.05). This trend was present at smaller tissue volumes, although the values at these volumes were not significantly different.

## Discussion

Flows ceased during apnea (Fig. 1). The recordings in Figure 1 show that, during apnea (tracheal obstruction), tracheal pressures cycled between −15 and −25 cm H_2_O while chamber pressures cycled between −25 and −35 cm H_2_O. In other words, lung inflation pressure (*P*_trachea_–*P*_chamber_) remained at 10 cm H_2_O: (−15–(−25)) = 10 cm H_2_O, and (−25–(−35)) = 10 cm H_2_O during apnea. This equaled the pulmonary artery pressure because the meniscus in the perfusion reservoir was set 10 cm above the base of the lung. Thus, during apnea, the lung was at the Zone 1–2 border. We believe this is the reason that perfusion stopped during apnea. Following apnea, after inflation pressures returned to preapnea levels, perfusate flowed from the lung at an increased rate for several seconds. This could not have been due to a rise in pulmonary artery pressure because the height of the perfusate reservoir remained unchanged during apnea. We believe it was due to perfusate accumulation on the arterial side of the microcirculation during apnea. The zone 1–2 border conditions during apnea apparently compressed the microcirculation enough to prevent flow through it. Once that compression was released, the accumulated perfusate began to flow through the microcirculation at an increased rate until the accumulation was relieved.

Significantly more particles entered the venous outflow from Control lungs than did so from Apnea lungs (Fig. 5, inset). Furthermore, particle trapping patterns within Control lungs were significantly less maldistributed than those in Apnea lungs, as shown by the more uniform particle distributions in confocal montages of those lungs (Fig. 4), and by the significantly lower logDI values (Fig. 5).

Based on these observations, we believe that some portions of the microcirculation in the Apnea lungs must have remained compressed after the fifth (last) apnea episode, when the latex particles were infused. This would explain why fewer particles were found in the venous effluent of Apnea lungs compared to Controls. If some microvessels remained compressed in the Apnea lungs when the particles were infused, the particles would not have had access to those vessels and would have been become trapped elsewhere. This would explain both the appearance of the particles in these lungs (Fig. 3) as well as the larger logDI value for them (Fig. 4).

It is interesting to note, however, that flows following particle infusion (Fig. 2) were not significantly different between Apnea and Control lungs. Even though particle trapping was less uniform in Apnea lungs than in Controls, it had no effect on perfusion through the Apnea  lungs.

We recently proposed that the vessels that allow 4 *μ*m diameter particles to enter the venous outflow are the acinar vessels that surround the alveoli (Watson et al. 2018). The vast majority of the infused 4 *μ*m particles remain trapped within those vessels, but the few particles that do enter the venous outflow appear to do so via those vessels (Conhaim et al. 2019a). Despite infusing 10^8^4 *μ*m particles into them, perfusate flows through the lungs remained unchanged. The only way this could be possible is if the acinar vessels are organized as a parallel perfusion network surrounding the alveoli, as we have previously proposed (Watson et al. 2018; Conhaim et al. 2019a). In such a network, obstruction of any vessels by microthrombi, including latex particles, would simply result in flow proceeding via unobstructed parallel routes throughout the network. This parallel network is so extensive that the flow remains unchanged despite the very large number of infused particles trapped within it.

Apnea affects these vessels in a way that disturbs particle trapping, perhaps because some of them remain closed even after inflation pressures have been returned to preapnea levels. This would also explain why fewer particles entered the venous outflow compared to Control lungs (Fig. 3) even though the particles were infused after the last apnea event. If this is so, it implies that a subset of acinar vessels were closed when the particles were infused. All are closed during apnea, which explains why perfusion stops. Once apnea ends and normal inflation pressures are restored, a subset of acinar vessels slowly reopen. This would explain the maldistributed particle trapping pattern, and the reduced numbers of particles in the venous outflow.

We recently proposed that the acinar vessels are more compressible than the vessels that supply them (Conhaim et al. 2019a). Our 4 *μ*m particle trapping data and our venous particle concentration data both support this idea.

### Hypoxia/hypercapnia

Hypoxia has been shown to raise pulmonary arterial pressure in OSA (Schneider et al. 2000). OSA is also associated with hypercapnia (Dempsey et al. 2010). Both could potentially influence pulmonary microvascular perfusion in intact animals. However, our isolated apnea and control lungs were not blood perfused, nor were they receiving systemic venous blood from metabolizing tissue. It therefore seems unlikely that either hypoxia or hypercapnia was responsible for the perfusion maldistribution revealed by the altered particle trapping patterns in our isolated, perfused lungs. We showed several years ago that exposure to 10% oxygen for 30 min in awake, intact rats did not alter particle trapping patterns within their lungs (Conhaim et al. 2008). This is further evidence that hypoxia was unlikely to have been responsible for the perfusion maldistributions we observed in our present studies.

It is more likely that the maldistribution was caused by the subatmospheric inflation pressures combined with the tracheal obstruction during simulated apnea. These forces were enough to obstruct pulmonary vessels and stop lung perfusion (Fig. 1). After apnea, some of the vessels were apparently not patent. This would explain the altered particle trapping patterns in apneic lungs into which particles were infused at the end of apnea. We found a similar phenomenon in the lungs of anesthetized rats subjected to apnea that was simulated by clamping the tracheas of these rats during spontaneous breathing. LogDI values in those lungs were larger (particles more maldistributed) in both Apnea and Control groups than those in our present isolated lung studies. In our anesthetized rat studies, we found that the apnea‐induced perfusion maldistribution was nearly gone if we waited 10 min after apnea before infusing the latex particles. This implies that the effects of the apnea on pulmonary microvascular perfusion distribution were temporary. It also implies that some of the vessels obstructed by apnea may have recovered more slowly than others, so that by 10 min, the effects of the apnea on microvascular perfusion distribution were nearly gone. In our isolated lung studies reported here, the effects of apnea on particle distribution might have also been reduced if we waited several minutes after apnea before infusing the latex particles. This idea will be the subject of future studies. An advantage of isolated lung studies is that we can measure venous latex particle concentrations, which is not possible in intact animal studies.

In summary, we found that simulated apnea caused microvascular perfusion maldistribution in isolated rat lungs, and also reduced the number of 4 *μ*m particles that were able to flow through those lungs. We believe that the subatmospheric pressures associated with the apneas together with a clamped trachea were responsible for these changes. We cannot rule out hypoxia as a source of perfusion maldistribution in intact animals. However, our present studies suggest that the deeply negative intrapleural pressures during apnea are responsible for perfusion maldistribution in isolated rat lungs. They may also be a factor that contribute to perfusion maldistribution in intact rats (Conhaim et al. 2019b).

## Conflict of Interest

None declared.
